# Antineoplastic with DNA fragmentation assay and anti-oxidant, anti-inflammatory with gene expression activity of *Lactobacillus plantarum* isolated from local Egyptian milk products

**DOI:** 10.1186/s12866-024-03576-y

**Published:** 2024-10-29

**Authors:** Mohamed A. Elhalik, Alsayed E. Mekky, Mohamed Khedr, Waleed B. Suleiman

**Affiliations:** Department of Botany and Microbiology, Faculty of Science, Al–Azhar University, Nasr City, Cairo, 11884 Egypt

**Keywords:** Antineoplastic, Antioxidant, Anti-inflammatory, DNA fragmentation, *Lactobacillus plantarum*

## Abstract

Many lactic acid bacteria (LAB), known for their human health benefits, are derived from milk and utilized in biotherapeutic applications or for producing valuable nutraceuticals. However, the specific role of milk-associated LAB in biotherapeutics remains underexplored. To address this, eight milk product samples were randomly selected from the Egyptian market, diluted, and then cultured anaerobically on MRS agar. Subsequently, 16 suspected LAB isolates were recovered and underwent rapid preliminary identification. Among these isolates, the *Lactobacillus plantarum* strain with accession number (OQ547261.1) was identified due to its strong antioxidant activity depending on the DPPH assay, *L. plantarum* displayed notable antioxidant activities of 71.8% and 93.8% at concentrations of 125–1000 µg/mL, respectively. While ascorbic acid showed lower concentrations of 7.81, 3.9, and 1.95 µg/mL which showed activities of 45.1%, 34.2%, and 27.2%, respectively. The anti-inflammatory efficacy of *L. plantarum* was evaluated based on its capability to prevent hemolysis induced by hypotonic conditions. At a concentration of 1000 µg/mL, *L. plantarum* could reduce hemolysis by 97.7%, nearly matching the 99.5% inhibition rate achieved by the standard drug, indomethacin, at an identical concentration. Moreover, *L. plantarum* exhibited high hemolytic activity at 100 µg/mL (14.3%), which decreased to 1.4% at 1000 µg/mL. The abundance of phenolic acids and flavonoids was determined by high-performance liquid chromatography (HPLC) in *L. plantarum*. Real-time quantitative reverse transcription polymerase chain reaction (qRT-PCR) demonstrated that *L. plantarum* increased gene expression of the inflammatory marker TLR2 by 133%, and cellular oxidation markers SOD1 and SOD2 by 65% and 74.2%, respectively, while suppressing CRP expression by 33.3%. These results underscore *L. plantarum’s* exceptional anti-inflammatory and antioxidant activities. Furthermore, *L. plantarum* induces cancer cell death through necrotic nuclear DNA fragmentation. These findings suggest that *L. plantarum* is not only suitable for nutraceutical production but also holds potential as a probiotic strain. Future research should focus on enhancing the capacity of this strain across various industries and fostering innovation in multiple fields.

## Introduction

Probiotics, when incorporated into food, contribute to a balanced microbial environment in the gastrointestinal tract, thereby supporting overall health. Research indicates that probiotics offer several health benefits, including antiallergic, and anticancer properties, a reduction in cholesterol levels, enhancement of the immune response, and alleviation of symptoms associated with irritable bowel syndrome and gastrointestinal inflammation [1]. The used probiotic strains are often derived from the gastrointestinal tract (GIT) itself or other sources, such as feces and milk. Lactic acid bacteria (LAB) are predominantly utilized in creating probiotic formulations. LAB, a natural part of the GIT flora in animals, has been deemed safe by the Food and Drug Administration [2]. Lactic acid bacteria (LAB) play a role in triggering immune responses and reducing or inhibiting the growth of pathogens through various mechanisms. Notably, numerous studies have identified their ability to inhibit α-glucosidase [3, 4]. LABs are also known for producing bacteriocins, protein substances capable of acting against pathogenic bacteria and possessing unique systems for breaking down different digestive substances [5]. Hernández-González et al. [6] explored the potential of LAB as immunomodulators, probiotics, and antimicrobials in veterinary applications. Additionally, research has consistently demonstrated that probiotics can act as antimicrobial agents, providing a viable alternative to traditional antibiotics. This antimicrobial effect, antagonistic to Gram-negative and Gram-positive pathogenic bacteria such as *E. coli*, *P. aeruginosa*, and *S. aureus*, contributes to the fight against antibiotic resistance [7].

*Lactobacillus plantarum* is a lactic acid bacterium (LAB) that is facultative heterofermentative. It can derive energy from various sugars and thrives in adaptable, nutrient-rich environments such as the gastrointestinal, vaginal, and urogenital tracts, as well as in vegetables, dairy products, and fermented foods. This bacterium possesses important characteristics that contribute to the production of a wide range of food and wine, as well as the production of vitamins, bacteriocin [8, 9], probiotic, antifungal, and potential anticaries agents [10]. *L. plantarum* 13 − 3 is characterized by its nonpathogenic nature, possessing 218 subsystems and 32,918 genes, as well as five classes of sugars that serve various essential functions [11]. Three strains of *Lactobacillus plantarum*, namely 12–3, K25, and YW11, were isolated from Tibetan Kefir. Various validated assays were conducted to assess their probiotic properties and antioxidant activity. *L. plantarum* 12–3 exhibited the highest activity in the DPPH radical scavenging test, with an average scavenging percentage of 58.1% ± 3.4%. The subsequent most effective strains were *L. plantarum* YW11 (52.3 ± 2.6%) and *L. plantarum* K25 (43.9 ± 2.6%). Based on these results, L. plantarum 12–3 displayed superior DPPH radical scavenging capabilities compared to the other two strains. Furthermore, L. plantarum 12–3 also demonstrated the most potent activity in the ABTS radical scavenging test, with an average scavenging percentage of 72.6 ± 2.9% [12]. The most common genetic material among the three L. plantarum strains played a role in carbohydrate metabolism, energy production and conversion, amino acid metabolism, and transcription [13]. However, an overabundance of ROS can lead to a multitude of pathological conditions, including DNA damage, carcinogenesis, and cellular degeneration, potentially triggering diseases like cancer, inflammation, lung injury, and other disorders [14, 15].

LAB strains have shown remarkable properties, such as wound healing and anti-inflammatory properties [16]. The demethylation of the TLR2 gene promoter has been linked to increased expression of pro-inflammatory cytokines and angiogenic markers, which are critical in the early stages of inflammation [17]. The initial discovery and documentation of TLR2, along with TLR1, TLR3, TLR4, and TLR5, occurred in 1998. Subsequent research over the years has highlighted TLR2’s pivotal role in vertebrate immune responses [18]. Unique among the Toll-like receptors (TLRs), TLR2 can form functional heterodimers with various other TLRs.

Moreover, TLR2 can engage with numerous non-TLR entities, facilitating the detection of a wide array of pathogen-associated molecular patterns (PAMPs) from all microbial domains, including fungi, viruses, parasites, and bacteria. The TLR2 gene is expressed not only by immune cells but also by endothelial and epithelial cells, underscoring its extensive involvement in immune responses [19]. In addition to its well-documented functions, inflammation is central to the pathogenesis of many diseases, with C-reactive protein (CRP) being a sensitive marker for inflammation. Recent findings suggest that CRP not only marks but also actively contributes to inflammatory and immune processes [20]. This emerging understanding of CRP’s role encompasses two immunological aspects: the initiation of the classical complement pathway via C1q binding, and the enhancement of immunity through the opsonization of biological particles and their interaction with Fcγ receptors on globulins [21].

The study aims to investigate the biotherapeutic potential of the *Lactobacillus plantarum* strain as a sustainable alternative to antibiotics and to explore its antioxidant and anti-inflammatory properties. Also, the study aims to assess the strain’s capacity to produce beneficial metabolites (nutraceuticals), its ability to induce cancer cell death via necrotic nuclear DNA fragmentation, and its potential as a probiotic strain across various industries. Additionally, the research aims to contribute to the understanding of milk-associated lactic acid bacteria (LAB) as biotherapeutics, further encouraging innovation in the field.

## Materials and methods

### Collection of milk samples

In the winter of 2022, eight different milk product samples were collected from the Egyptian market using sterile glass tubes. These samples were then transported to the laboratory in containers that maintained a controlled temperature and were stored at 4 °C until they were ready for experimentation.

### Isolation of *Lactobacillus* spp. from samples

The milk samples were first diluted with saline, then pour-plated onto MRS agar, and incubated in an anaerobic environment at 37 °C for a period ranging from 24 to 48 h. Following incubation, colonies that appeared morphologically distinct and well-separated were chosen for further cultivation. These selected colonies were then subcultured onto new MRS agar plates using the streak plate method to obtain pure cultures [22].

### The first identification of the *Lactobacillus* strains

Identifying the pure cultures involved utilizing Bergey’s Manual of Determinative Bacteriology and software tools such as PIBWin and IDENTAX [23, 24]. An in-depth examination of the colonies’ physical attributes was conducted, including observations of their shape, color, and texture.

Subsequently, the isolates underwent gram staining and were examined under a microscope to verify their purity. Isolates identified as gram-positive rods that were also catalase-negative underwent further characterization. This included tests for cytochrome oxidase activity, ability to grow at temperatures of 15 °C and 45 °C, and acid production from various carbohydrates like L-arabinose and D-fructose, among others, in designated media. The characterization process also encompassed evaluating acid and gas production from glucose, performing methyl red and Voges-Proskauer tests, assessing ammonia production from arginine, and testing for nitrate reduction. Finally, the pure isolates were evaluated by incubating them in MRS broth enriched with NaCl of 2% to select the best organisms that recorded high absorbance.

### Molecular identification of the most potent *Lactobacillus strain* amplification and sequencing of 16S rRNA

The genomic DNA from LAB isolates were isolated using the QIAamp DNA Mini Kit (Qiagen SA, Courtaboeuf, France). The concentration and purity of the DNA were assessed using a NanoDrop spectrophotometer, followed by PCR amplification with two universal bacterial primers for the 16S rRNA gene: 27 F (5′-AGAGTTTGATCCTGGCTCAG-3′) and 1492R (5′-TACGGYTACCTTGTTACGACTT-3′) [25]. The PCR products were then purified with the QIAquick PCR Purification Kit (Qiagen SA, Courtaboeuf, France) and sequenced on an ABI 3500 Genetic Analyzer (Applied Biosystems, Foster City, CA, USA).

The 16S rRNA gene sequences were analyzed using Geneious Bioinformatics software (Version 11, available at http://www.geneious.com). The gene sequence was further compared using the Basic Local Alignment Search Tool at the National Center for Biological Information (NCBI), where they were submitted to obtain accession numbers [26, 27]. Phylogenetic tree construction was carried out following protocols described in previous studies, using the Tamura genetic distance model, the neighbor-joining method for tree construction, and validating the tree with one thousand bootstrap resamples. The phylogenetic tree was visualized by GeneDoc and Geneious software tools.

### Study of the optimization parameters for increasing the growth yield of *Lactobacillus plantarum*

#### Effects of different incubation periods and incubation conditions

This experiment aimed to determine the best incubation time for cultivating a highly effective bacterial strain on MRS medium, which is tailored for lactic acid bacteria. The bacterial strain was incubated for different intervals (0, 10, 20, 30, 40, and 50 h), under both static conditions and with agitation at 150 rpm. Following each incubation interval, the optical density of the culture was assessed using a 721 spectrophotometer (M-ETCAL) [28].

#### Effects of different temperatures

The pure culture of *Lactobacillus* isolate was suspended in MRS broth and incubated at 20, 25, 30, 35, 40, 45, and 50 °C for 48 h. Growth, indicated by turbidity, was evaluated after 48 h for the higher temperatures. This approach assessed the bacterial isolates’ growth capabilities at different temperatures [28].

#### Effects of different pH values

The optimal components of MRS media were adjusted to a range of pH levels (2, 3, 4, 5, 6, 7, 8, and 9) using a buffer solution to mitigate pH changes caused by metabolic processes. The optical density of the culture was measured after their respective incubation periods [28].

#### Effects of different nitrogen sources

The effect of different organic and inorganic nitrogen sources, as well as amino acids such as ammonium nitrate, ammonium sulfate, urea, peptone, tryptophan, and yeast extract, on growth was evaluated with an equivalent nitrogen level present in each medium used. All other optimal conditions were maintained as previously described [29].

#### Effects of different carbon sources

MRS media for lactic acid bacteria were each enriched with various carbon sources, each at a 0.5% concentration. These carbon sources included sucrose, glucose, maltose, starch, lactose, and bagasse. All the previously mentioned optimal conditions were applied in each instance. After every incubation period, the optical density was measured using a spectrophotometer (721 spectrophotometer, M-ETCAL) [29].

#### NaCl tolerance test

The tolerance to sodium chloride (NaCl) of the isolate was evaluated by incubating it in MRS broth enriched with varying concentrations of NaCl (2%, 3%, 4%, 5%, 6%, 7%, and 8%). After adding 10 ml of the overnight culture of the isolate to the broth, the samples were then incubated anaerobically at 37 °C for 18 to 24 h. The growth of *Lactobacillus plantarum* was determined by measuring the absorbance at 600 nm using MRS broth without NaCl as the control [30].

#### Bile salt tolerance test

The evaluation of bile tolerance was conducted as described by Jomehzadeh et al. [30]. *Lactobacillus plantarum* was initially grown overnight at 37 °C in MRS broth. For the bile tolerance test, these cultures were then introduced into MRS broth tubes containing 0.3% (w/v) bile salts (Oxgall) at a 1% (v/v) concentration. The inoculated tubes were incubated at 37 °C for 2, 4, 6, and 8 h. Tubes that were not inoculated acted as controls for the experiment. Bacterial growth was measured using a spectrophotometer, recording the optical density (O.D.) at 660 nm.

#### Ethanol tolerance test

For the ethanol tolerance assessment, the isolate was cultured in MRS broth containing different ethanol concentrations (2.5%, 5%, 10%, 15%, and 20%) [31]. The bacterial suspension was prepared in the same manner as described previously, with 100 µL transferred into 10 mL of various MRS broth. After incubation, 1 mL was taken from each broth and diluted appropriately. Subsequently, the total viable cell counts were determined using the plate count method. Each experiment was performed in triplicate.

#### Estimation of active compounds produced by *L. Plantarum*

The HPLC investigation was done using an Agilent 1260 series. The division was completed by utilizing Zorbax overshadowing. Furthermore, the mobile phase consisted of water (A) and 0.05% trifluoroacetic acid in acetonitrile (B), utilized alongside the C8 column (4.6 mm x 250 mm, 5 μm) at a flow rate of 0.9 ml/min. The portable stage was modified continuously on a straight slope as follows: 0 min (82% A); 0–1 min (82% A); 1–11 min (75% A); 11–18 min (60% A); 18–22 min (82% A); 22–24 min (82% A). The multi-frequency finder was observed at 280 nm. The infusion volume was 5 µL for every one of the example arrangements. The section temperature was kept at 40 °C.

### In vitro assessments of *L. plantarum*

#### Antineoplastic activity of *L. plantarum* using the MTT assay

Cell metabolic activity was evaluated using the 3-(4,5-dimethylthiazol-2-yl)-2,5-diphenyl tetrazolium bromide (MTT) assay from Sigma-Aldrich, following the protocol previously described [32]. The purpose of this assessment was to ascertain the effect of bacterial supernatants on the metabolic processes of HCT 116 cells, independent of cell proliferation. Initially, a consistent monolayer of HCT 116 cells was prepared as described earlier. These cells were then exposed to incremental concentrations of CFS, CFSp, and CFSpe for 24–48 h in an environment composed of 5% CO_2_ and 95% air. Cells were incubated in the standard complete medium (DMEM with 10% FBS) and acted as reference controls.

The optical density (OD) was recorded at 490 nm. The results were calculated using the equation: OD of treated cells at each time point / OD of reference control at T0 × 100 [33].

### Measurement of DNA fragmentation

DNA fragmentation was assessed using the method described by Sugihara et al. [34]. Briefly, cells weighing 25 mg were homogenized and washed with a phosphate buffer solution (PBS) that included 10 mM EDTA. The cell lysis was carried out with 250 ml of lysis buffer (pH 8.0), comprising 10 mM Tris base, 1 mM EDTA, and 0.2% Triton X-100, followed by incubation at -20 °C for 20 min. After incubation, the lysates were centrifuged at 10,000 rpm for 15 min at 4 °C to separate the intact chromatin from the fragmented DNA (supernatant). The pellet was dissolved in 0.5 N perchloric acid, while 5.5 N perchloric acid was added to the supernatant to achieve a final concentration of about 6.0 N. The samples were then heated at 90 °C for 20 min and centrifuged at 10,000 rpm for 10 min to remove residual proteins. To each sample, 160 ml of diphenylamine (DPA) solution [comprising 150 mg DPA in 10 ml glacial acetic acid, 150 ml sulfuric acid, and 50 ml acetaldehyde (16 mg/ml)] was added, followed by incubation at room temperature for 24 h. The absorbance at 600 nm was measured using a UV double-beam spectrophotometer (Shimadzu, Tokyo, Japan). The percentage of DNA fragmentation was calculated with the formula: %DNA fragmentation = (OD of supernatant / (OD of supernatant + OD of pellet)) x 100, where OD represents optical density [35].

### DNA assay through gel electrophoresis

The assessment of DNA fragmentation was carried out by analyzing the nuclear DNA laddering pattern, following the method outlined by Majtnerova et al. [36]. In detail, cells were first homogenized and incubated overnight at 37 °C in phosphate-buffered saline (PBS). This step was followed by cell lysis in 0.5 mL of DNA extraction buffer, which contained 50 mM Tris-HCl, 10 mM EDTA, 0.5% Triton, and 100 mg/mL proteinase K at a pH of 8.0. Afterward, the lysate was treated with 100 mg/mL DNase-free RNase at 37 °C for 2 h. Subsequently, the mixture underwent three sequential extractions with an equal volume of chloroform and phenol (1:1 v/v), and then one more extraction with chloroform alone, with each extraction followed by centrifugation at 13,000 rpm for 5 min at 4 °C. DNA was then precipitated by adding two volumes of ice-cold absolute ethanol and one-tenth volume of 3 M sodium acetate, pH 5.2, and the mixture was left to stand at -20 °C for 1 h before being centrifuged at 10,000 rpm for 10 min at 4 °C. The DNA pellet was rinsed with 70% ethanol, air-dried, and dissolved in 10 mM Tris-HCl and 1 mM EDTA, pH 8.0. The isolated DNA was then subjected to electrophoresis in a 1.5% agarose gel and stained with ethidium bromide, using Tris/acetate/EDTA (TAE) buffer (pH 8.5, 2 mM EDTA, and 40 mM Tris-acetate) for visualization [33].

### Anti-inflammatory activity

#### Preparation of erythrocyte suspension

Workers’ fresh whole blood (3 ml) was drawn into heparinized tubes and centrifuged at 3000 rpm for 10 min to separate the components. The red blood cell (RBC) pellets obtained were resuspended in an equal volume of normal saline, matching that of the previously removed supernatant. This step was followed by 40% (v/v) suspension of the resuspended RBCs in an isotonic phosphate buffer solution (10 mM sodium phosphate buffer, pH 7.4), calculated according to the adjusted volume. The isotonic buffer solution was formulated by dissolving 0.2 g of NaH_2_PO_4_, 1.15 g of Na_2_HPO_4_, and 9 g of NaCl in 1 l of distilled water. This buffered solution was used to facilitate the application of the reconstituted red blood cells (the resuspended supernatant).

#### Hypotonicity-induced hemolysis

In this study, extracts were dissolved in distilled water to create a hypotonic solution [37]. For each concentration level (100, 200, 400, 600, 800, and 1000 g/ml), 5 ml of this solution containing the extracts was placed into pairs of centrifuge tubes. Similarly, varying concentrations of the extracts, from 100 to 1000 g/ml, were added to pairs of centrifuge tubes filled with 5 ml of an isotonic solution. As controls, tubes were prepared with 5 ml of a 200 g/ml indomethacin solution and 5 ml of distilled water, serving as the vehicle. To each tube, 0.1 ml of an erythrocyte suspension was added and then gently mixed. The tubes were incubated for an hour at room temperature (37 °C) and centrifuged at 1300 g for 3 min. The optical density (OD) reflecting the concentration of hemoglobin in the supernatant was measured at 540 nm using a Spectronic (Milton Roy) spectrophotometer. Assuming distilled water causes 100% hemolysis, the percentage inhibition of hemolysis by the extracts was calculated using the formula: % Inhibition of hemolysis = 1 - ((OD2 - OD1) / (OD3 - OD1)) * 100. Here, OD1 represents the absorbance of the test sample in the isotonic solution, OD2 represents the absorbance in the hypotonic solution, and OD3 represents the absorbance of the control sample in the hypotonic solution.

#### Hemolytic assay

Fresh human blood was collected and washed thrice with 150 mM NaCl (at 2500 rpm for 10 min). The plasma was discarded, and the cells were resuspended in phosphate-buffered saline (PBS, pH 7.4) to achieve a 2% RBC concentration. Serial two-fold dilutions of the extract (concentrations of 1000, 800, 600, 400, 200, 100, and 50 µg/ml) were mixed with a 2% RBC solution, and the total volume of the reaction mixture was adjusted to 1 ml with PBS. The reaction mixture was incubated in a water bath at 37 °C for 1 h. Following incubation, the mixture was centrifuged at 2500 rpm for 15 min. The optical density of the collected supernatant was measured at 541 nm, using PBS as the blank [38]. Deionized water served as a positive control. The experiment was performed in triplicate, and the results were expressed as mean ± S.D. The percentage of hemolysis was calculated using the formula: percentage hemolysis = [(Absorbance of sample - Absorbance of blank) × 100] / Absorbance of positive control.

### Antioxidant activity

The antioxidant capacity of *L. plantarum* was assessed using the DPPH (2,2-diphenylpicrylhydrazyl) free radical scavenging method [39, 40]. Briefly, a solution of 0.1 mM DPPH in ethanol was prepared. To each 1 ml of this solution, 3 ml of the extracts at varying ethanol concentrations (from 3.9 to 1000 g/ml) were added. For this investigation, only ethanol-soluble extracts were utilized, and diluted to different concentrations. After thorough mixing, the solutions were allowed to stand at room temperature for 30 min. The absorbance was recorded at 517 nm using a UV-VIS Milton Roy spectrophotometer. Ascorbic acid served as the reference standard, and the procedure was repeated three times. The IC_50_ value, indicative of the concentration needed to reduce 50% of the DPPH free radicals, was calculated from the log dose inhibition curve. A reduction in the absorbance of the reaction mixture correlates to an increase in free radical scavenging activity. The percentage of DPPH inhibition was calculated using the formula: inhibition (%) = [Absorbance of control - Absorbance of sample) / Absorbance of control] × 100.

### Total RNA isolation and cDNA synthesis

Total RNA was isolated from 48-hour cultures of LAB strains grown in liquid MRS medium using the Trizol reagent (Gibco) according to the protocol provided by the manufacturer. The synthesis of the first-strand cDNA was performed using the Advantage RT-for PCR Kit (Clontech, Palo Alto, CA, USA). Reverse transcription polymerase chain reaction (rt-PCR) was executed in 50 µL reactions using specific primers detailed in Table 1. Each PCR reaction mix included 75 ng of the generated cDNA, 200 mM dNTPs, 0.1 mM of each specific primer, 1.5 mM MgCl2, and 1 U of Taq DNA polymerase (Takara), with the final volume adjusted to 50 µL with sterile water. The PCR amplification followed these conditions: an initial denaturation step at 94 °C for 4 min; 32 cycles of denaturation at 94 °C for 30 s, annealing at 50 °C for 50 s, extension at 71.5 °C for 1 min; and a final extension at 71.5 °C for 5 min. The PCR products were then run on an agarose gel using TAE buffer (40 mM Tris-Acetate, pH 7.6, and 1 mM EDTA). After electrophoresis, the gels were stained with ethidium bromide (0.5 mg/ml), and the bands were visualized under UV light. The lengths of the amplicons were compared to a standard DNA marker (GeneRuler™ 100 bp DNA Ladder, MBI Fermentas, Vilnius, Lithuania) to determine their sizes [41].

**Table 1:** PCR-specific primers which used to amplify four genes beside RecA as a housekeeping gene

Primer	Sequence (5’->3’)	Length	Tm	GC%	Strand	Ref
FW primer- TLR2	GCTCAGACTTGAGCACTATACA	22	57.62	45.45	+	**This study**
RW primer- TLR2	GGCTTGAACCAGGAAGACGA	20	59.9	55	-	
FW primer 1- CRP	CGACCCGTGGGTACAGTATTT	21	59.7	52.3	+	
RW primer 1- CRP	TAACGAGCTCCCAGACCAGA	20	59.9	55	-	
FW primer 2- CRP	ACAGTTTTACAGTGGGTGGGT	21	59.4	47.6	+	
RW primer 2- CRP	TTGCTGGGCTTCCCATTTCA	20	60.1	50	-	
FW primer 4- SOD1	ACTTGGGCAATGTGACTGCT	20	60.1	50	+	
RW primer 4- SOD1	TGGGCGATCCCAATTACACC	20	60.1	55	-	
FW primer 9- SOD1	CGTGGCCTAGCGAGTTATGG	20	60.6	60	+	
RW primer 9- SOD1	ATAGACACATCGGCCACACC	20	59.8	55	-	
FW primer 3- SOD2	GTTCCGAGTTTTCCAGGCAC	20	59.4	55	+	
RW primer 3- SOD2	CACCTGAAGTCAAGTGGGCT	20	59.8	55	-	
FW primer 6- SOD2	GGTTTTGGGGTATCTGGGCT	20	59.6	55	+	
RW primer 6- SOD2	ATCGTGCCTGGAAAACTCGG	20	60.6	55	-	
FW primer 7- SOD2	TGGAGGAGAACTCGCTTCGT	20	60.8	55	+	
RW primer 7- SOD2	CCCCAAAAGGCACAGACTCA	20	60.1	55	-	
FW 1–18 S	GCCCTAATTGGTCCAGGCG	19	44	44.3	+	
RW 1–18 S	ACAACGGCGTTCTCTCCTAT	20	44	44.4	-	
FW 2–18 S	ACACAACGTCATTGCAAATGTGA	23	44	44.3	+	
RW 2–18 S	GCCTGGACCAATTAGGGCAT	20	44	44.3	-	

### Real-time PCR amplification conditions

Using the primers shown in Table 1, complementary DNA (cDNA) from six samples (three treated and three controls) was subjected to semiquantitative PCR. 2x Quantitech SYBR^®^ Green RT Mix (Fermentase) containing approximately 12.5 l of the 25 l of the real-time PCR experiment consisting of 1 l of 50 ng cDNA, 1 l of 25 pm/l from each forward and reverse primer, and 9.25 l of RNase-free water. The centrifuge was used on the samples before they were loaded into the rotor wells. PCR program set for interest genes as starting denaturation for 2 min at 95 °C, then cycles at 95 °C/30 sec, annealing at 57.5 °C/30 sec, extension step at 72 °C/30 sec. On the other hand, the PCR program for 18 S, which is a standard housekeeping gene, was optimized and adjusted as follows: denaturation at 95 °C/2 min, annealing at 44 °C/25 sec, and extension at 72 °C/30 sec. The Rotor Quality 6000 gear from Qiagen in the USA was utilized to do the response. The PCR cycle at the threshold cycle (CT) results are present [42].

### Data analysis

Using the dd∆ct technique and Microsoft Excel, a comparative quantification analysis was carried out.

## Results and discussion

Probiotics, recognized for their nutraceutical benefits, require verification of their safety for human health before they can be endorsed as beneficial gut bacteria. The market is replete with various probiotic strains, presenting an opportunity to utilize this readily available resource for isolating and crafting new, improved probiotic strains with significant medical benefits. This approach is a promising strategy for the development of superior probiotic strains [43]. The genus *Lactobacillus* represents a broad and varied collection of gram-positive, non-sporulating, facultatively anaerobic bacteria. It includes species such as *Lactobacillus plantarum*, *Lactobacillus fermentum*, *Lactobacillus paracasei*, *Lactobacillus acidophilus*, *Lactobacillus rhamnosus*, *Lactobacillus bulgaricus*, *Lactobacillus casei*, and *Lactobacillus reuteri*, among others. This sort assumes an imperative part in food maturation and can likewise be viewed as in the gastrointestinal (G arrangement of people and creatures in factor sums [44]. Eight examples of milk were randomly collected from the markets in Egypt. The initial samples were anaerobically plated onto MRS agar after being serially diluted.

**Fig. 1:**
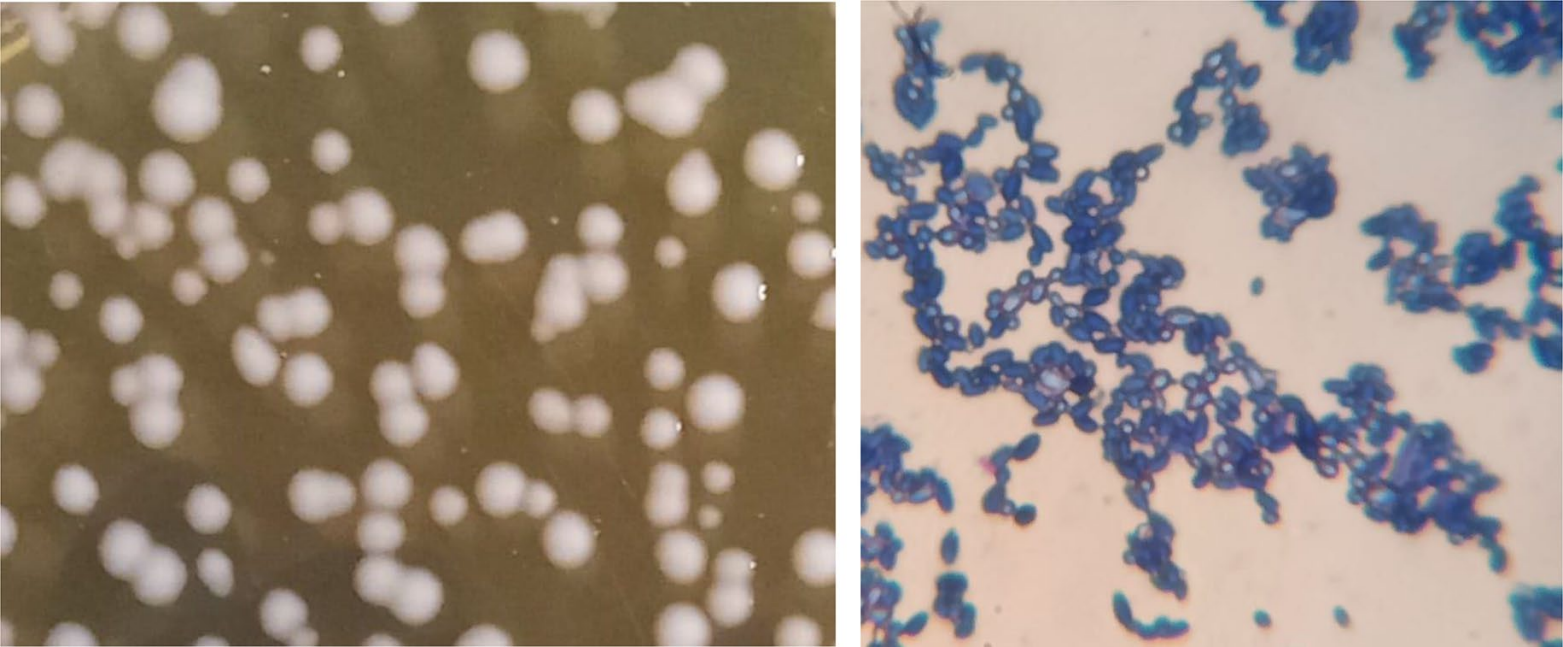
The morphological colonies of *Lactobacillus* sp. on MSR medium and its shape under light microscope. (X:1500)

A total of 16 lactic acid bacteria isolates were retrieved from the previously mentioned samples and subjected to rapid preliminary identification. The total discoveries of morphological and biochemical tests. The best organism has been selected based on its ability to grow at a 2% salt concentration and yield the highest absorbance. The morphological and biochemical tests of the best organism are introduced in Table 2. Staining revealed the gram-positive nature of the isolate, which had a purple or violet color. The isolate was rod-shaped and had long, rounded ends. They showed up generally as a chain of 3–4 cells, either single or two by two (Fig. 1). The hanging drop strategy showed that the microscopic organisms were non-motile, which is one of the interesting attributes of *Lactobacilli*. This may be because of the shortfall of extraordinary propeller-like flagella in *Lactobacilli* answerable for motility. Similar findings were made by Forouhandeh [45] in the isolation of *Lactobacillus* species from various dairy products. The absence of air bubbles indicated that the isolated microbes were catalase-negative, thereby incapable of breaking down hydrogen peroxide to release oxygen. It is well established that *Lactobacillus* species do not produce catalase. Comparable findings were documented by Mithun et al. [46].

**Fig. 2:**
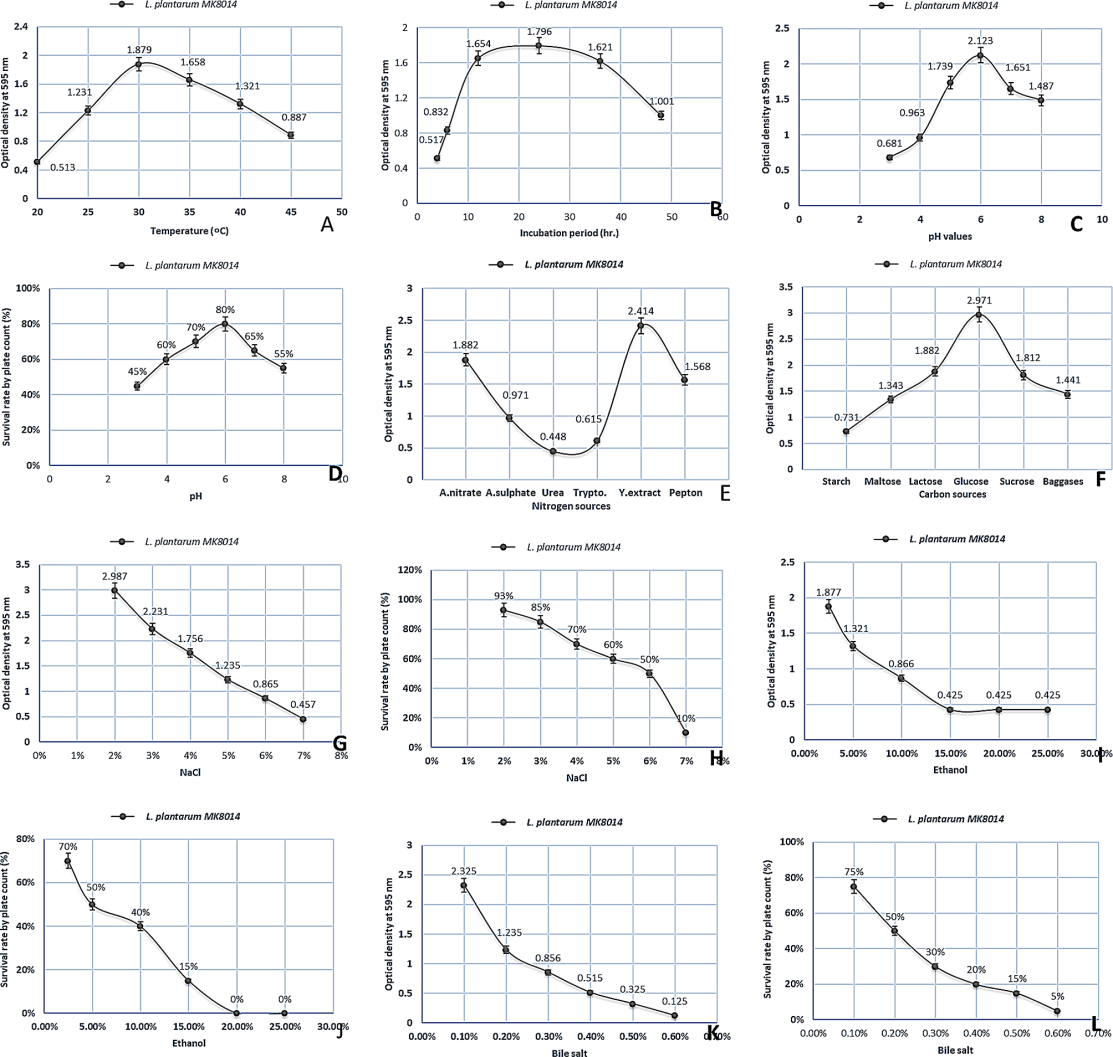
Effect of different (**A**) Temperatures, (**B**) Incubation periods, (**C**) PH, (**D**) PH (%), (**E**) Nitrogen sources, (**F**) Carbon sources, (**G**) NaCl, (**H**) NaCl (%),(**I**) Ethanol, (**J**) Ethanol (%),(**K**) Bile salt, (**L**), Bile salt (%) on the growth viability of *L. plantarum* strain isolate

Preliminary optimization, including pH, temperature, incubation period, carbon, nitrogen sources, NaCl, ethanol, and bile salt, was used in this study. The *L. plantarum* strain was incubated at varying temperatures, from 20 ºC to 45 ºC. The findings indicated that the optimal growth temperature for the *L. plantarum* isolate was 30 °C, with an enzyme activity level of 1.879 ± 0.130. Conversely, the lowest enzyme activity, 0.513 ± 0.01, was observed at a lower temperature of 20 ^o^C, with enzyme activity diminishing sharply as temperatures approached this lower limit. Consequently, the study identified that enzyme activity decreased at temperatures below 30 °C, while increasing up to 35 ^o^C, establishing 30 ^o^C as the ideal temperature for cultivating the *L. plantarum* strain (Fig. 2 (A)). The best incubation duration for producing the *L. plantarum* strain was detected at 24 h, with the most conducive conditions for its growth under static conditions being 1.796 ± 0.012 (Fig. 2 (B)). The study also explored the effect of pH levels on the production of the *L. plantarum* strain, revealing that the most favorable pH for its production was 6, with an optimal yield of 2.123 ± 0.27 (80%) (Fig. 2 (C&D). Furthermore, the research investigated the impact of different carbon and nitrogen sources on the strain’s production. It was found that glucose combined with yeast extract resulted in the highest production levels, with values of 2.971 ± 0.020 and 2.414 ± 0.26, respectively (Fig. 2 (E and F)). The optimal production of the *L. plantarum* strain was achieved using lactose as the carbon source and ammonium nitrate as the nitrogen source. Additionally, the study assessed the tolerance of the *L. plantarum* strain to NaCl, ethanol, and bile salts, recording values of 2.987 ± 0.2 (93%) at 2% (Fig. 2 (G&H)), 1.877 ± 0.2 (70%) at 2.5% (Fig. 2 (I&J)), and 2.325 ± 0.2 (75%) at 0.1% (Fig. 2 (K&L)), respectively, demonstrating the strain’s resilience to these conditions. Similarly, Coulon et al. [47] have reported that *Lactobacillus casei* ATCC393 found 35 ^o^C to be the optimal temperature for the production of β-glucosidase among the various temperature ranges. Also, the ideal temperature for compound creation from yeast, such as *Debaryomyces pseudopolymorphus* [48] and *Saccharomyces cerevisiae* [49], was 40 °C, respectively. The ideal pH for *Lactobacillus rhamnosus* CRL 98 was 6.4, which was like the *L. plantarum* strain, which expressed that various upsides of ideal pH were accounted for in various types of lactic corrosive microscopic organisms. *Lactobacillus mesenteroides* [50] and *Lactobacillus plantarum* [51] grow at an optimum pH of 5.0 and 5.4, respectively. The metabolism of carbon sources releases energy, which is utilized by the organism for its growth and development. The influence of different carbon sources on the production of *L. plantarum* strains was investigated in this study. It was discovered that lactose serves as an efficient carbon source for cultivating the *L. plantarum* strain. Interestingly, the organism demonstrated significant growth on xylose, resulting in the highest protein activity in the culture broth compared to other carbon sources tested. This observation indicates that the *Lactobacillus plantarum* strain can thrive or adapt to diverse environmental conditions. In certain examinations of glycosidase chemicals from different sources, Grimaldi et al. [52] have announced that the presence of glucose or fructose diminishes the exercises. Also, when the culture in a fluid medium contained corn grain, the greatest aggregate β-glucosidase creation was accounted for, cellulose and glucose prompted elevated degrees of β-glucosidase creation, *Pediococci* sp. creation of β-D-glucopyranosidase movement was unequivocally worked on by both glucose and fructose. It was presented as an expected supportive source from which higher compounds could be cleansed [53]. *L. plantarum* strain was developed on carbon wellsprings of focus 1% m/v.

In many microorganisms, both organic and inorganic nitrogen forms are metabolized to produce amino acids, nucleic acids, proteins, and components of the cell wall [54]. It was found that the use of ammonium nitrate resulted in the highest production levels of the *Lactobacillus plantarum* strain among various organic and inorganic nitrogen sources tested, with the urea-containing medium showing the lowest activity. Regarding the impact of different ethanol concentrations on the optical density and viability of the *L. plantarum* strain, it was observed that a 2.5% (v/v) ethanol concentration yielded the highest activity compared to other tested concentrations. Furthermore, Spano et al. [55] reported that the β-glucosidase gene from *Lactobacillus plantarum* was inhibited by 12% (v/v) ethanol [52]. Notably, ethanol was found to activate the enzyme in wine at concentrations above 15% (v/v).

In contrast, Grimaldi et al. [52] noted that the stimulatory effect of 4% v/v ethanol is more pronounced at lower concentrations, aligning with previous findings for *Oenococcus oeni* and other yeast biomasses. The study led with various groupings of NaCl for the creation of *L. plantarum* showed the greatest worth at a convergence of 2% NaCl. It has been demonstrated that sodium ions in the surrounding environment are necessary for efficient membrane transport. The utilization of NaCl in a concentrate by Damaso et al. [56] demonstrated that it is necessary for the synthesis of another enzyme, xylanase.

**Fig. 3:**
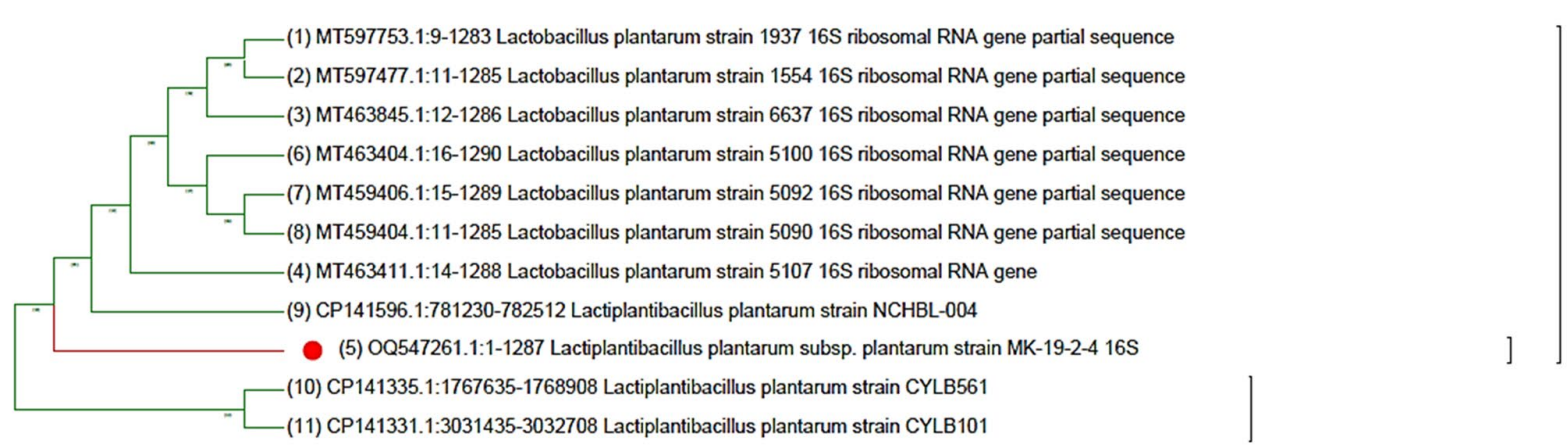
Phylogenetic tree of gene sequences of *L. plantarum* isolate with the sequences retrieved from the NCBI GenBank site

*Lactobacilli* was identified genetically through PCR amplification and sequencing of 16S rDNA using two bacterial universal primers, as mentioned. The PCR amplicon was purified from the gel, then sequenced and aligned through NCBI BLASTn for firmly related sequences on the NCBI GenBank database; this indicates that it belonged to *Lactobacillus plantarum*, and its phylogenetic tree was designed through MEGA 11 with the most related sequences as described in Fig. 3. This technique was used by Khedr et al. [41] to identify *Lactobacillus delbrueckii*. Likewise, Abdel Ghany et al. [42] used this technique to identify *Lactobacillus acidophilus*.

**Table 2:** The morphological and biochemical characterization of the best organism

No.	Characteristics	Results
**Morphological tests**		
1	Gram staining	+
2	Shape	Rod
3	Colony morphology	Circular, white, glistering, convex.
4	Motility	Non motile
**Biochemical tests**		
1	Catalase	-
2	Citrate test	-
3	NH3 from arginine	-
4	Hydrogen sulfide production	-
5	Indole production	-
6	Methyl red reaction	+
7	Oxidase test	-
8	Urease test	-
9	Voges Proskauer Reaction	-
10	Glucose utilization	+
11	Glucose (Gas) (Co_2_)	-
12	Sucrose utilization	+
13	Lactose utilization	+
14	Maltose utilization	+
15	Mannitol utilization	+
16	Arabinose utilization	-
17	Salicin utilization	+

+, positive result; -, negative result

High-Performance Liquid Chromatography (HPLC) is a critical and versatile analytical method extensively employed across various fields. It is utilized for both the separation and quantification of organic and inorganic substances in a wide array of samples, including those from industrial, pharmaceutical, food, and environmental sources. Moreover, it is increasingly applied to biological samples and the extraction of natural products. Through HPLC, compounds are differentiated based on their interactions with the mobile phase solvent and the solid material within a tightly packed column, under high pressure. This technique is gaining recognition as a preferred method for fingerprinting analysis, crucial for the quality assurance of herbal products [57]. Various researchers and scholars have highlighted the application of HPLC in the characterization and quantification of secondary metabolites in plant extracts, specifically targeting phenolic compounds, steroids, flavonoids, and alkaloids [58–60].

Flavonoids constitute a vast group of polyphenolic compounds, all structurally derived from the base molecule flavone and produced by plants. These substances, found in fruits and vegetables, are recognized for their broad and significant health benefits, which include radical scavenging and metal-chelating activities. The antioxidant properties of flavonoids in vitro stem from their ability to mitigate free radical formation, leading to various biological effects. Numerous studies have highlighted that flavonoids such as rutin, kaempferol, quercetin, and apigenin, among others, are renowned for their anti-inflammatory, anti-allergic, antithrombotic, hepatoprotective, antispasmodic, and anticancer properties [61, 62]. The biological activities, such as their antimutagenicity, antibacterial, antiviral, anti-inflammatory, and apoptotic properties, among others, must be justified by distinguishing and quantitating such mixtures [63]. Syringic acid is accepted to have various advantageous natural exercises, including the insurance of the mind, heart, and liver, as well as anticancer, antimicrobial, against aggravation, antidiabetic, hostile to nitrosative, and cell reinforcement properties [64]. Coumaric acid demonstrates a wide range of bioactive properties, including antioxidant, anti-inflammatory, anti-mutagenic, anti-ulcer, antiplatelet, and anti-cancer activities. Besides, it plays a role in mitigating atherosclerosis, oxidative damage to the heart, damage to ocular tissues caused by UV light, neuronal injury, anxiety, gout, and diabetes [65]. According to the current study, CA may alleviate diabetes by increasing its immunomodulatory effect and defending against oxidative stress and inflammation. Quercetin has drawn expanding consideration because of its cancer prevention agent, and antibacterial, and anti-inflammatory effects [66, 67].

**Fig. 4:**
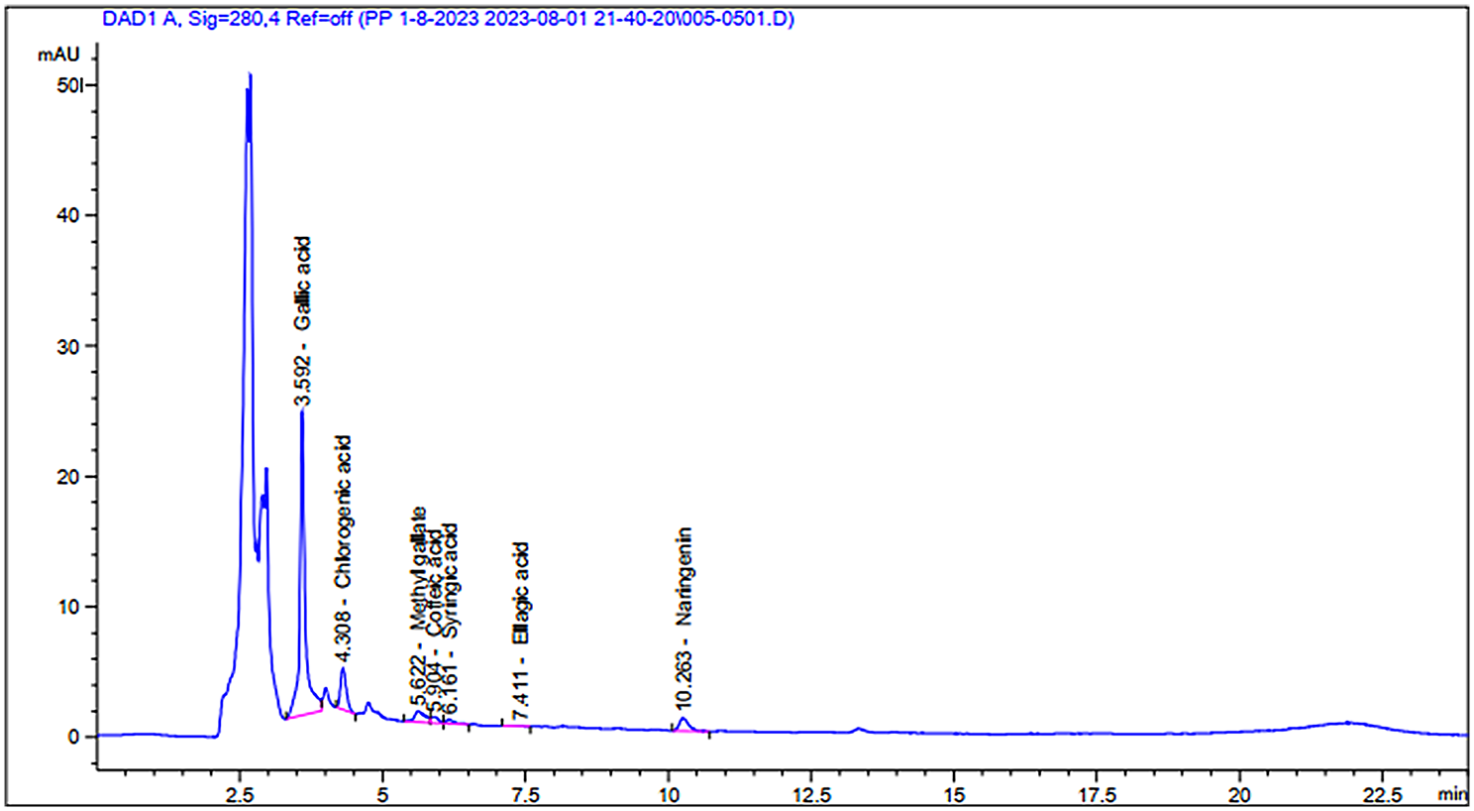
HPLC profile of bioactive compounds produced by *L. plantarum*

The results from the study on *L. plantarum* indicate that the strain tested can produce secondary metabolites with promising antimicrobial, antioxidant, and anti-inflammatory properties (Table 3; Fig. 4). However, to validate *L. plantarum’s* potential as a biomedical agent, in vivo studies will be necessary.

**Table 3:** Bioactive compounds isolated from *L. Plantarum*

Peak No.	RT	Type	Area %	Compound name	Molecular weight (g/mol)	Molecular formula
1	3.592	BV	74.5124	Gallic acid	170.12	C_7_H_6_O_5_
2	4.308	BB	10.7387	Chlorogenic acid	354.31	C_16_H_18_O_9_
3	4.494	-	0.0000	Catechin	290.26	C_15_H_14_O_6_
4	5.622	BV	5.2960	Methyl gallate	184.147	C_8_H_8_O_5_
5	5.904	VV	2.0099	Caffeic acid	180.16	C_9_H_8_O_4_
6	6.161	VB	3.1779	Syringic acid	198.17	C_9_H_10_O_5_
7	6.649	-	0.0000	Pyro catechol	110.1	C_6_H_6_O_2_
8	6.925	-	0.0000	Rutin	610.517	C_27_H_30_O_16_
9	7.411	BB	0.4946	Ellagic acid	302.197	C_14_H_6_O_8_
10	8.702	-	0.0000	Coumaric acid	*164.16*	*C9H8O3*
11	9.123	-	0.0000	Vanillin	152.15	C_8_H_8_O_3_
12	9.756	-	0.0000	Ferulic acid	194.18	C_10_H_10_O_4_
13	10.263	BB	5.4176	Naringenin	272.257	C_15_H_12_O_5_
14	11.846	-	0.0000	Rosmarinic acid	360.318	C_18_H_16_O_8_
15	16.021	-	0.0000	Daidzein	254.23	C_15_H_10_O_4_
16	17.331	-	0.0000	Quercetin	302.236	C_15_H_10_O_7_
17	19.263	-	0.0000	Cinnamic acid	148.1586	C_9_H_8_O_2_
18	20.610	-	0.0000	Kaempferol	286.23	C_15_H_10_O_6_
19	21.205	-	0.0000	Hesperetin	302.27	C_16_H_14_O_6_

### Antineoplastic activity of *Lactobacillus plantarum*

**Fig. 5:**
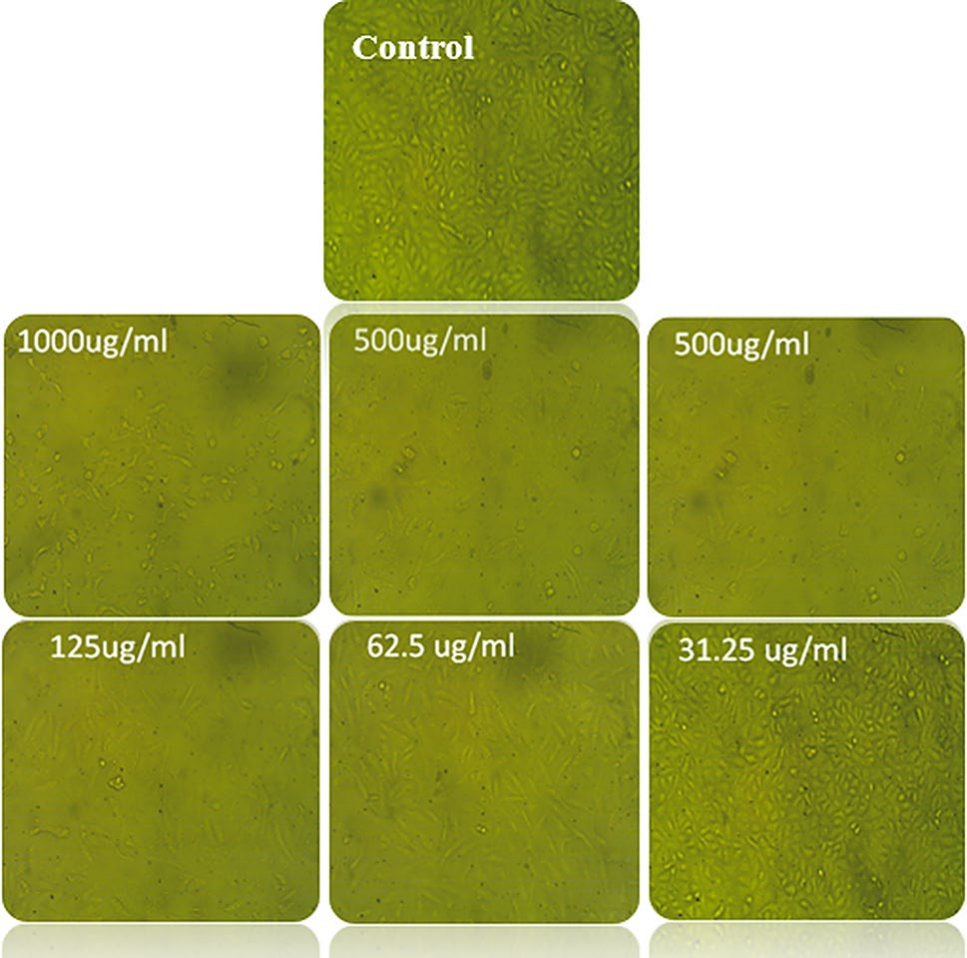
Morphological characteristics of cancer cells (HCT116) treated with *L. plantarum*

Figure 5 illustrates changes in cell morphology and shape within a monolayer culture as an initial and distinct effect observed following exposure to *L. plantarum* captured using an inverted light microscope. The inhibitory effect of *L. plantarum* on human colon cancer cells (HCT116), along with the degree of cell suppression, was confirmed using the MTT assay at different concentrations ranging from 1000 to 31.25 µg/mL. Significantly, the IC50 value, indicating the concentration needed to inhibit 50% of the cancer cells (HCT116), was found to be 100.11 µg/mL. *Lactobacillus* strains are commonly recognized for their health-promoting roles as microbial food supplements, with benefits such as enhancing gut health, boosting the immune system, and lowering the risk of certain cancers [68]. Regular consumption of yogurt and other probiotic dairy products has been suggested to inhibit the growth of colon cancer cells [69]. The surface components of *Lactobacillus* strains have shown anticancer activities. This study explores the anti-cancer capabilities of *L. plantarum*. Previous research has indicated the role of autophagy in cancer prevention and treatment. This investigation found distinctive morphological and biochemical markers of autophagy, such as autophagic vacuoles and acidic vesicular organelles, in HCT116 colon cancer cells treated with *L. plantarum*, suggesting that *L. plantarum* triggers autophagic cell death in HCT116 colon cancer cells [70].

### DNA fragmentation ability of *Lactobacillus plantarum*

**Fig. 6:**
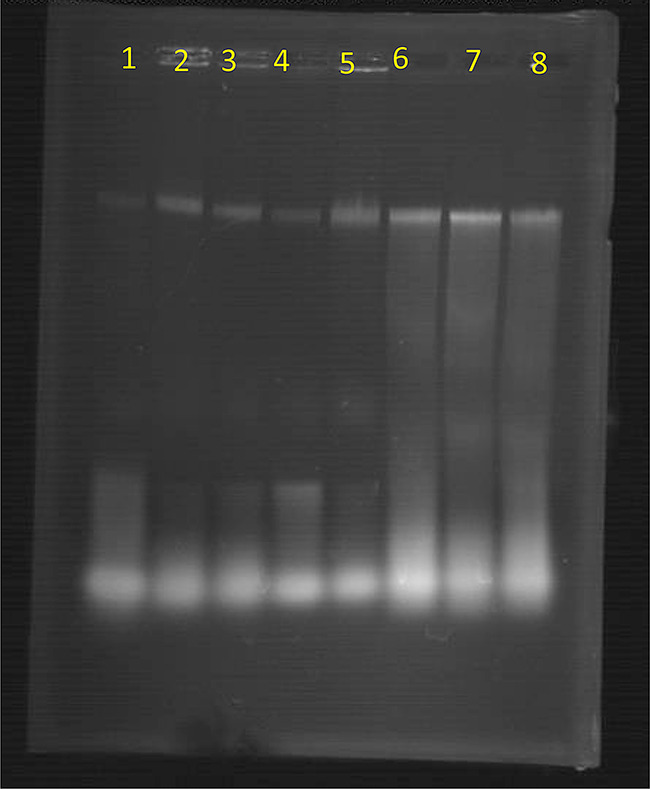
Gel electrophoresis of nuclear DNA of untreated and treated cancer cell lines with *L. plantarum*, where lanes 1, 2, 3 and 4 are for control cancer cells and lanes 5, 6, 7 and 8 are for treated cells

Our research demonstrates that *L. plantarum* significantly contributes to triggering cell death in cancer cells via DNA fragmentation, indicative of necrosis. This observation is supported by gel electrophoresis findings, which displayed DNA fragmentation in cells treated with *L. plantarum*, in stark contrast to the intact DNA in untreated (control) cancer cells, as illustrated in Fig. 6. This finding differs from the behavior of nuclear DNA in cancer cells. Moreover, Choi et al. [71] discovered that soluble polysaccharides from the cell wall of *L. acidophilus* 606 inflicted damage on HT-29 cancer cells, a phenomenon largely attributed to the initiation of apoptosis rather than necrosis, as evidenced by nuclear DNA fragmentation and the lack of PI staining. This marks a pioneering instance of cancer cell apoptosis triggered by *Lactobacilli*-derived polysaccharides.

Additionally, proteomic analysis revealed that polysaccharides from *L. acidophilus* 606 significantly affected the expression of proteins such as the Bcl-2-interacting mediator and cell division cycle proteins. These findings underline the potent antioxidative and anticancer properties of soluble polysaccharides from *L. acidophilus* 606 against various cancer cell lines. The potential of these polysaccharide components to be integrated into foods or used as supplements in cancer therapy is significant [72, 73]. Furthermore, *L. plantarum* is shown to modulate the expression of crucial genes like AKT, PTEN, BAX, and TLR4, which are involved in apoptosis and anti-apoptosis mechanisms in the AGS gastric cancer cell line [74].

### Anti-inflammatory activity of *Lactobacillus plantarum*

**Fig. 7:**
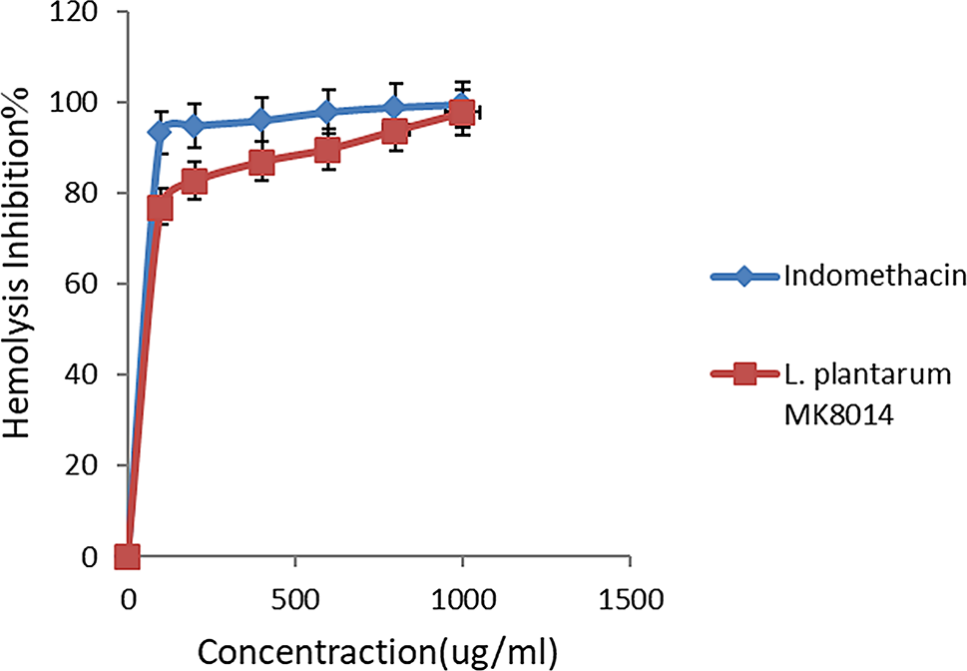
Effect of *L. plantarum* on HRBC hemolysis and membrane stabilization

**Fig. 8:**
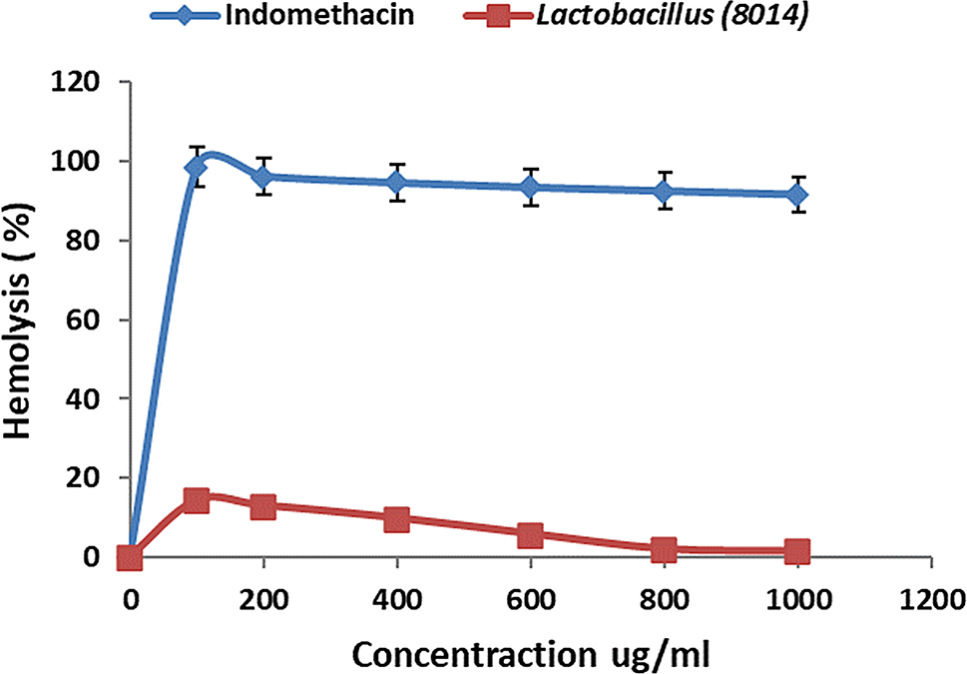
Effect of *L. plantarum* on hemolytic activity

The evaluation of *L. plantarum’s* anti-inflammatory effects was conducted through its capacity to suppress hypotonicity-induced hemolysis and perform hemolytic assays in vitro. The results showed that *L. plantarum* significantly reduced hemolysis by 97.7% at a concentration of 1000 µg/mL, nearly matching the 99.5% effectiveness of indomethacin, a widely recognized anti-inflammatory medication, at the same concentration (Fig. 7). On the contrary, the hemolytic activity of *L. plantarum* peaked at 14.3% at a concentration of 100 µg/mL and then diminished to 1.4% at 1000 µg/mL (Fig. 8). These findings underscore the potential of *L. plantarum* as an effective anti-inflammatory agent [75]. Similarly, *L. casei* and *L. acidophilus* have been observed to significantly alleviate paw swelling in rats, indicating their anti-inflammatory properties. Research conducted by Ganji-Arjenaki and Rafieian-Kopaei [76] has shown the efficacy of various *Lactobacillus* strains in the treatment of inflammatory bowel disease. In contrast, a study found that exopolysaccharides from *Bacillus circulans* exhibited a 92% anti-inflammatory effect, while EPS from *Pseudomonas mendocina* AB1 showed a lesser effect of 59.07% [77]. These comparative insights call for further investigation to elucidate the mechanisms of EPS in protein protection and their application in developing new anti-inflammatory treatments [78].

The *L. plantarum* strain we studied exhibited notable anti-inflammatory properties by reducing the expression of two critical markers of inflammation in human cells: CRP and TLR2. Specifically, the expression levels of CRP and TLR2 decreased by one and eight times, respectively, in the cell line treated with our strain, which was fermented for 72 h and then incubated for five hours at 37 °C. These findings are consistent with in vitro research conducted by Borchers et al. [79], which indicates that *Lactobacillus plantarum* 299v may reduce inflammation in humans through the suppression of TLR activation. Additionally, the interaction observed between human peripheral blood mononuclear cells (PBMCs) and *L. plantarum* species underscores *L. plantarum’s* potential to modulate PBMC responses [80].

### Antioxidant activity of Lactobacillus plantarum

**Fig. 9:**
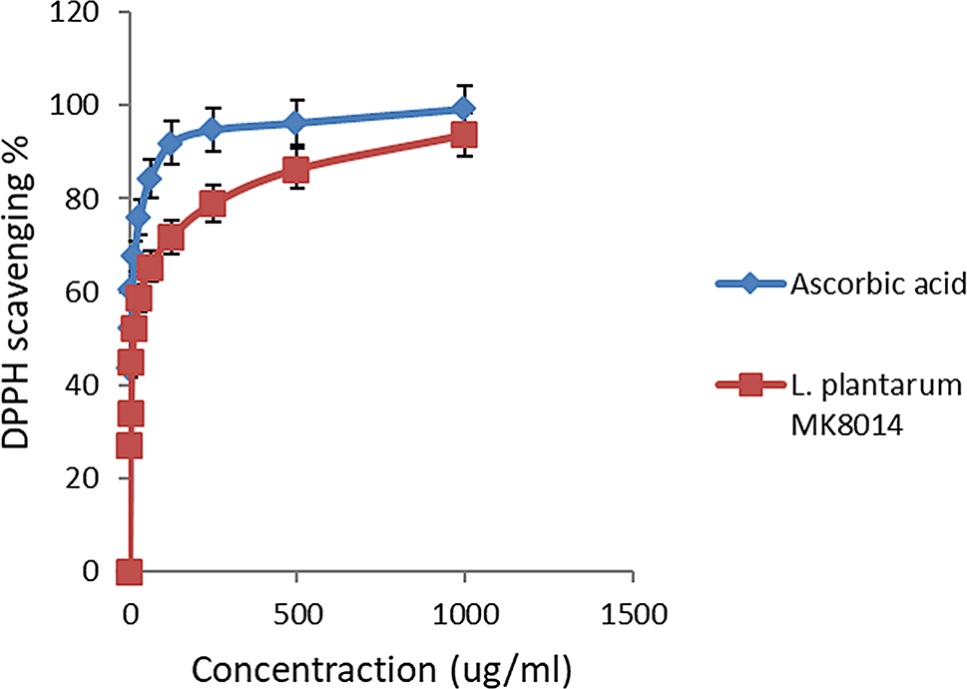
Antioxidant activities of *L. plantarum* using DPPH assay

Lactic acid bacteria (LAB), including those with antioxidant enzymes, are vital for enzymatic defense against oxidative stress. The antioxidant potential of *L. plantarum* was evaluated over a spectrum of concentrations from 1000 to 1.95 µg/mL, as shown in Fig. 9. The results indicated that *L. plantarum* exhibited notable antioxidant efficiency, with activities of 71.8% and 93.8% at concentrations ranging from 125 to 1000 µg/mL, respectively. However, at lower concentrations of 7.81, 3.9, and 1.95 µg/mL, the observed antioxidant activities were 45.1%, 34.2%, and 27.2%, respectively, when compared to ascorbic acid, the standard reference used. Moreover, *L. plantarum* has demonstrated its capacity to counteract free radicals. Our study highlights that *L. plantarum* strains AR113, AR269, AR300, AR501, and *P. pentosaceus* AR243 showed considerable resilience against hydrogen peroxide [81]. In this context, *L. plantarum* was recognized for its profound antioxidant activity. This is in line with the findings of Li et al. [82], who found that *L. plantarum* strains from traditional Chinese fermented foods possess antioxidant capabilities, with *L. plantarum* C88 showcasing optimal hydroxyl radical and DPPH scavenging activities against hydrogen peroxide at a density of 1010 CFU/ml. The DPPH scavenging efficiency of our isolates surpassed those documented by Benattouche et al. [83], who reported antioxidant activities ranging from 16 to 56% for exopolysaccharides derived from various yogurt LABs at a concentration of 1000 µg/mL.

### Gene expression was induced by *Lactobacillus plantarum*

**Fig. 10:**
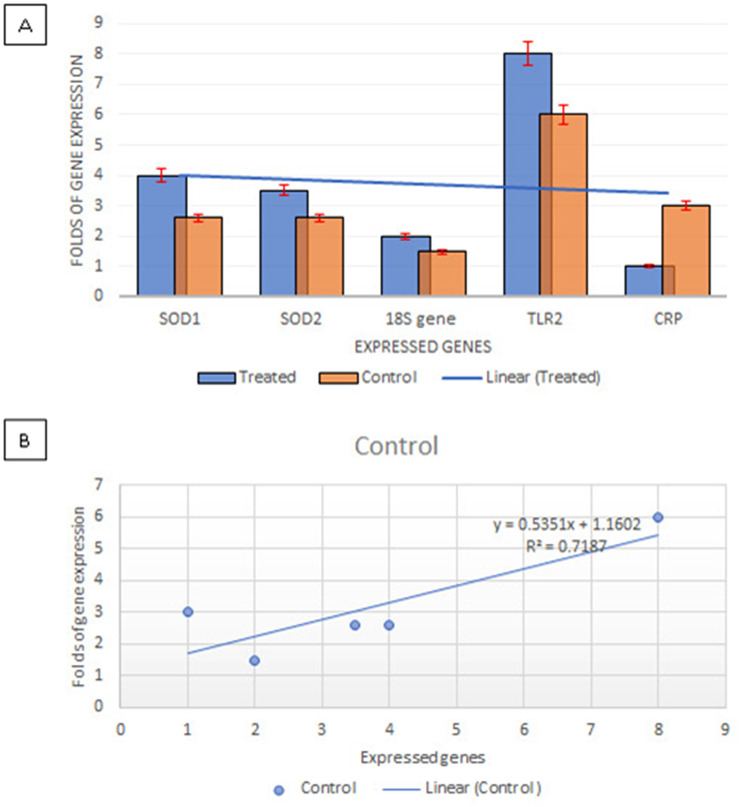
**A**, **B.** Folds of gene expression for four genes as anti-inflammatory markers TLR2 and CRP beside antioxidant markers SOD1 and SOD2 against control

Moreover, *L. plantarum* has been found to modulate the expression of the antioxidant markers SOD1 and SOD2, enhancing their levels by 65% and 74.2%, respectively. It also significantly boosts the gene expression of TLR2 by 133% compared to the control, while reducing CRP expression by 33.3%, as depicted in Fig. 10A and B. These results are in line with findings by Rolfe [84], who observed that LAB supplementation could mitigate oxidative stress in piglets. Many probiotics, particularly LAB, are increasingly recognized as alternatives to antibiotics and as therapeutic options for managing post-weaning syndrome. They achieve their beneficial effects through various actions, including immune system activation, pathogen invasion blockade, and antimicrobial substance production. Supplementation with LAB notably enhances (*p* < 0.05) the expression of Btk, HO-1, Nrf2, TLR4, and TLR2 in the jejunum, in contrast to the LPS-only group. Protein expression of TLR4, Btk, and Nrf2 in the ileum of LPS-challenged piglets was also elevated (*p* < 0.05) following LAB supplementation [85]. Additionally, LAB helps shield the intestine from oxidative damage in animals by activating antioxidant enzymes and preserving redox homeostasis [86].

**Fig. 11:**
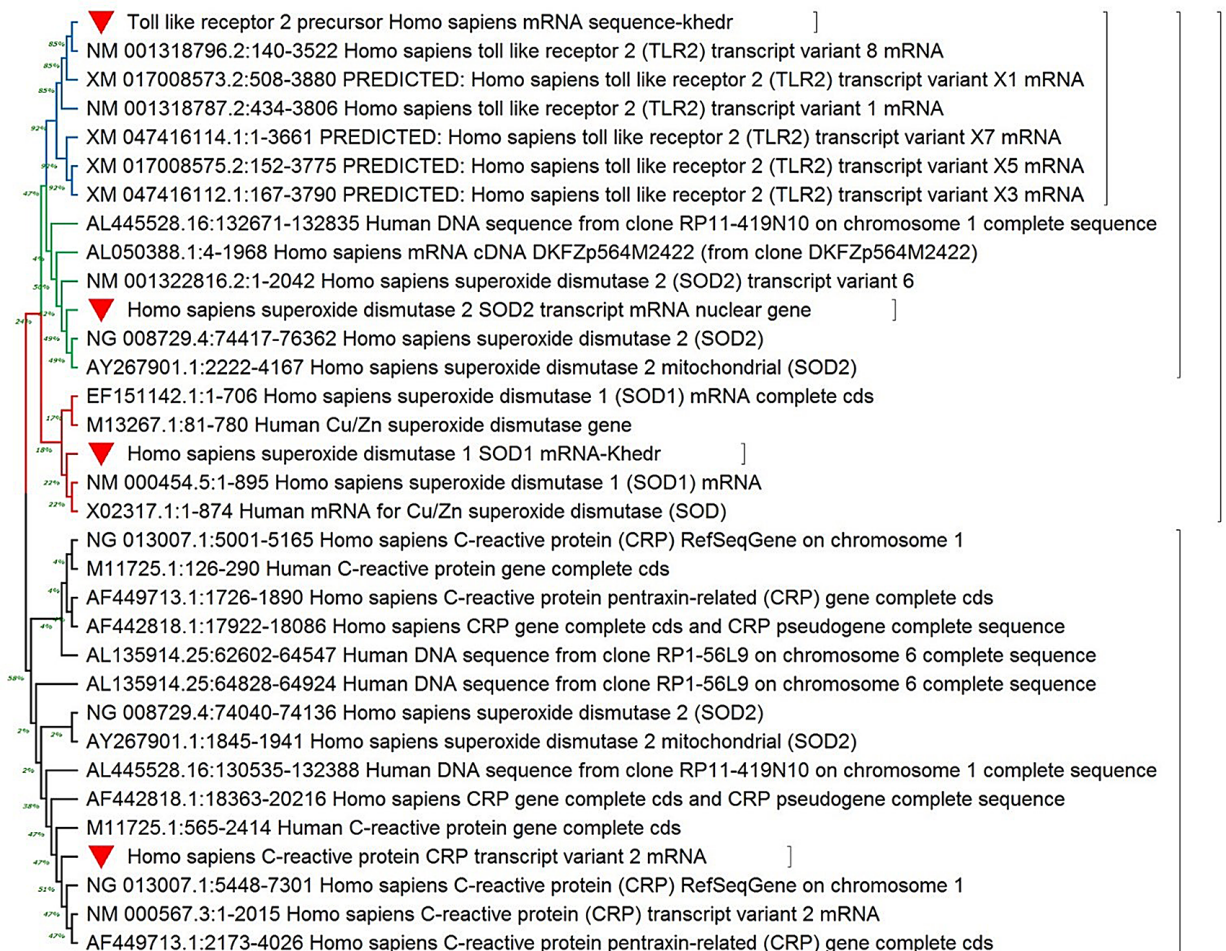
Phylogenetic tree of four interest genes, A: CRP, B: TLR2, C: SOD1, and D: SOD2

Four interest gene sequences were aligned against the most related sequences in the NCBI database; based on their sequences, phylogenetic trees were constructed as shown in Fig. 11.

## Conclusion

Our strain *Lactobacillus plantarum* (OQ547261.1) is a local isolate from milk products in the Egyptian market with a rich content of phenolic acids and flavonoids which was demonstrated by HPLC. It successfully showed inspiring antioxidant activity and a promising anti-inflammatory activity. Moreover, RT-qPCR analysis demonstrated that *L. plantarum* significantly increased expression of inflammation (TLR2) and oxidation markers (SOD1 and SOD2) while reducing CRP expression. Interestingly, *L. plantarum* also induced necrotic cancer cell death, highlighting its potential in nutraceuticals and as a valuable probiotic strain, suggesting a need for further research to explore its broad applications.

## Data Availability

All datasets generated throughout this research are included in the manuscript. Regarding 16S rDNA of Lactobacillus plantarum, the sequence was submitted to GenBank on NCBI as a member of INSDC repository. Accession numbers are available on: https://www.ncbi.nlm.nih.gov/nuccore/OQ547261.1.
